# Supplementary material for: Altered expression of mRNA profiles in blood of early-onset schizophrenia
Source: Sci Rep. 2016 Jan 6;6:16767. doi: 10.1038/srep16767 (PMC4702094; doi:10.1038/srep16767)
Supplement: Supplementary Information [file srep16767-s1.doc]

**Altered expression of mRNA profiles in blood of early-onset schizophrenia**

Yong Xu^1, #^, Yin Yao Shugart^2, #^, Guoqiang Wang^3^, Zaohuo Cheng^3^, Chunhui Jin^3^, Kai Zhang^3^, Jun Wang^3^, Hao Yu^4,5^, Weihua Yue^4,5,*^, Fuquan Zhang^3,*^ & Dai Zhang^4,5,6^

^1^Department of Psychiatry, First Hospital /First Clinical Medical College of Shanxi Medical University, Taiyuan, China

^2^Unit on Statistical Genomics, Division of Intramural Research Program, National Institute of Mental Health, National Institutes of Health, Bethesda, Maryland, United States of America

^3^Wuxi Mental Health Center, Nanjing Medical University, Wuxi, Jiangsu Province, China

^4^Institute of Mental Health, Sixth Hospital, Peking University; Beijing 100191, China.

^5^Key Laboratory of Mental Health, Ministry of Health & National Clinical Research Center for Mental Disorders (Peking University), Beijing, 100191, China.

^6^Peking-Tsinghua Center for Life Sciences / PKU-IDG/McGovern Institute for Brain Research, Peking University, Beijing, 100871, China.

^#^ These authors contributed equally to this work

Corresponding Author:

E-mail address: F.Z. ([zhangfq@njmu.](mailto:zfqeee@126.com)edu.cn); W.Y. (dryue@bjmu.edu.cn)

**Abstract**

To identify gene expression abnormalities in schizophrenia (SZ), we generated whole-genome gene expression profiles using microarrays on peripheral blood mononuclear cells (PBMCs) from 18 early-onset SZ cases and 12 controls. We detected 84 transcripts differentially expressed by diagnostic status, with 82 genes being upregulated and 2 downregulated. We identified two SZ associated gene coexpression modules (green and red), including 446 genes . The green module is positively correlated with SZ, encompassing predominantly up-regulated genes in SZ; while the red module was negatively correlated with disease status, involving mostly nominally down-regulated genes in SZ. The olfactory transduction pathway was the most enriched pathways for the genes within the two modules. The expression levels of several hub genes, including AKT1, BRCA1, CCDC134, UBD, and ZIC2 were validated using real-time quantitative PCR. Our findings indicate that mRNA coexpression abnormalities may serve as a promising mechanism underlying the development of SZ.

Key Words: schizophrenia; mRNA; coexpression; microarray

Schizophrenia (SZ) is a severe chronic mental disorder affecting about 1% of the population worldwide. Generally arising in late adolescence, it profoundly disrupts a few key traits of human cognition and personality including language, thought, perception, emotional affect, and sense of self. SZ patients tend to first present with overt symptoms during late adolescence or early adulthood and it has been postulated that this developmental stage represents a “window of vulnerability”. When the disease manifests before age 18, it is defined as early-onset SZ (EOS), a subcategory of SZ associated with more familial vulnerability and poor outcomes[^1^](#_ENREF_1).

Despite SZ’s wide prevalence and debilitating nature, little is known about its pathogenesis. Clinicians therefore tend to rely on clinical symptoms for diagnosis and for evaluating the progress and treatment response throughout the course of the disease. It has been hypothesized that the gene expression is the most fundamental level at which the genotypes critically influence the SZ phenotypes.

It is almost impractical to obtain biopsied brain tissue from SZ patients for the development of a molecular signature that may assist a diagnosis. In this regard, peripheral blood mononuclear cells (PBMCs) can be easily collected from patients and followed longitudinally with gene expression analyses, which may provide a way of identifying the signatures of clinical subtypes, their prognosis and treatment response.

To detect biomarkers for SZ and other psychiatric disorders, a few earlier studies profiled gene expression in peripheral blood[^2-7^](#_ENREF_2). However, the results varied across studies. To date, a consistent pattern of alterations has not been established.

The expressions of transcripts are influenced by many environmental factors, including medication. Most previous studies were conducted using patients under drug treatment or with a history of pharmacotherapy, which makes it impossible to preclude the potential effect of antipsychotic therapy. However, several studies have been conducted on PBMCs in drug -naive participants. Craddock et al.[^9^](#_ENREF_9) used blood T cell derived RNA and compared the gene expression from six minimally treated or first-episode SZ patients (age 31.6±14.1) with controls and identified 399 differentially expressed (DE) probes. Of these, 320 (80%) probes were decreased in SZ and 79 were increased. Takahashi et al.[^10^](#_ENREF_10) identified 792 DE probes, with 256 probes being downregulated and 536 probes (68%) being up-regulated in blood of antipsychotics-free SZ patients (age 31.8 ±11.4) compared with controls. Recently, Kumarasinghe et al.[^11^](#_ENREF_11) detected 416 (67%) down-regulated and 208 up-regulated genes in blood of treatment-naive patients (age 36.1±14.8) when compared to controls. However, these studies showed that the patients had diverse disease durations and their age spanned a wide range (>20 years), which will certainly affect the results. Furthermore, it remains controversial concerning the global direction of changes in genome expression for SZ.

It is well known that genes tend to work together to perform its function, and functionally related genes tend to be coexpressed. It is tempting to apply the gene coexpression network analysis to results interpretation. Gene coexpression networks encapsulate the activity of multiple regulatory systems and hold the potential to highlight specific molecular mechanisms for disease[^14^](#_ENREF_14). Differential coexpression refers to variations in gene–gene correlation between two sets of phenotypically distinct samples[^15^](#_ENREF_15). Gene–gene correlation may change without affecting differential expression, indicating that a gene may alter its regulatory pattern that would be missed by traditional differential expression analyses. Differential coexpression analysis (DCEA), which aims to find gene modules with different connectivity (correlations) in the disease state, offers a more powerful approach for elucidating transcriptome patterns and dysfunction of gene expression underlying phenotypic changes. Several studies conducted gene coexpression analysis for mental health disorders[^16-20^](#_ENREF_16). One commonly used method is weighted gene coexpression network analysis (WGCNA)[^21^](#_ENREF_21).

In this work, we aimed to identify potential biological markers for SZ using blood-based gene expression profiles from a cohort of EOS and healthy controls. We detected a panel of individually misexpressed genes implicated in SZ, suggesting an upregulation trend of gene expression in SZ. Further, we identified two clusters of coexpressed genes associated with SZ. Further analysis revealed intensive interactions among the genes within the two clusters and that several hub genes may represent new causal candidate genes for SZ.

**Results**

**mRNA profiles in SZ cases compared with controls**

In this study, we used the microarray platform and detected a total of 8594 mRNAs. Cluster analysis is shown in Supplementary Figure S1. DE mRNAs were identified through FC and P value filtering. With P values adjusted using Bonferroni correction (FC ≥ 2 and P_adjusted_< 0.05), 84 mRNAs were DE, of which, 82 were upregulated and 2 downregulated in SZ patients compared with healthy control subjects (Figure 1, Supplementary Table S1). Major GO categories of the 84 DE genes are shown in Supplementary Figure S2. Gene ontology analysis identified 10 categories with P value < 0.001, three of which reached FDR significance (receptor activity, plasma membrane, and nucleosome) (Figure 2, Supplementary Table S2). KEGG (Kyoto Encyclopedia of Genes and Genomes) pathway analysis identified 30 non-directional enriched pathways (Supplementary Table S3).

Assuming a sample size in each group of 15 and a FDR of 0.05, our dataset offers a power of 83.3% to detect DE genes (Supplementary Figure S3). Since our samples comprised of 18 cases and 12 controls, the power of our dataset may be slightly lower the estimated power

**Coexpression analysis**

Genes with coefficient of variation of expression levels less than 0.1 were filtered out. A total of 2484 out of 8518 genes were retained in the coexpression analysis. The cluster dendrogram is shown in Figure 3. There were 7 coexpressed modules, among which 2 modules (green and red) were significantly associated with SZ (Table 1), including a total of 446 genes (Supplementary Table S4). The green module is positively correlated with SZ, meaning that the genes in this module are predominantly up-regulated in SZ cases. In contrast, the red module was negatively correlated with disease status, involving mostly down-regulated genes in SZ. Topological Overlap Matrix (TOM) plot of the network connections is shown in Supplementary Figure S4, and gene significance across modules is shown in Supplementary Figure S5.

Notably, 55 out of 84 DE genes were among the 238 green module genes. All of the 55 genes were up-regulated in SZ compared with controls. Given that we observed a total of 2484 genes in the coexpression modules, we would like to indicate the strength of the statistical rigor for our finding (P < 2.2E-16).

Inner module structure of the green and red modules is shown in Figure 4. Genes with high intra-modular connectivity are informally referred to as intra-modular hub genes. Our results indicated that CCDC134 and UBD are hub genes in the green module. While within the red module, C10orf67, TNIP3, and ZIC2 are hub genes.

**Pathway analysis of the green and red module**

Protein–protein interaction (PPI) analysis shows the existence of complex interactions among the 446 genes within the green and red modules (Figure 5A). AKT1, CHEK1 and BRCA1 were hub genes in the network. GO analysis shows that 10 biological processes are enriched in the 446 module genes (Supplementary Table S5). KEGG-based pathway analysis indicates that olfactory transduction and protein digestion and absorption are two enriched pathways (Figure 5B).

**Real-time quantitative PCR (RT-qPCR) analysis of hub genes**

We evaluated blood expression levels of AKT1, BRCA1, CCDC134, UBD, and ZIC2 in 48 healthy controls, and 30 SZ patients before and after a 12-week following-up treatment using RT-qPCR. Our data show that GAPDH was expressed at a stable level across the samples. At the threshold of FDR < 0.05, AKT1 and BRCA1 were up-regulated in SZ cases compared with controls (Figure 6, Supplementary Table S6); while CCDC134, UBD, and ZIC2 were down-regulated after the 12-week treatment (Figure 6, Supplementary Table 7).

**Discussion**

The transcriptome sits between environmental influence and the genetic susceptibility to SZ and thus may serve as a bridge between certain endophenotypes and the genetic changes that lead to the disorder. To gain a better understanding of the neuropathology of SZ, therefore, we sought to conduct mRNA profile examinations in a cohort of drug-free teenaged patients with first-onset SZ. The study group was homogeneous in age, all were students, and had no other major physical diagnosis. Most importantly, all the patients were untreated, precluding any confounding effects of therapeutic drugs. Because of the fact that the patients were in the early stage of disease and given their ages, their brains were still developing. We therefore speculate that the alterations in transcription identified in these patient cohorts are likely to be relevant to the disease process.

The period of late adolescent development is of particular interest to psychiatry research, as this time window corresponds to the age of onset of major neuropsychiatric disorders, especially SZ. We focused specifically on this period, with the goal of identifying genes whose expression is altered during this period. We detected predominant up-regulation of mRNAs in the SZ group, with 82 out of 84 DE genes being increased. This finding was consistent with those of Takahashi et al.[^10^](#_ENREF_10) regarding the change direction. This observation seems valuable considering that transcript studies are often plagued with uncertainties, inconsistences and conflicts due to fluctuations of gene expression and a myriad influencing factors[^3^](#_ENREF_3).

Gene ontology analysis of the 84 DE genes showed that these genes were enriched in signal transduction, multicellular organismal development, metabolic process, ion transport, cell cycle, and cell differentiation. Among the 84 DE genes, solute carrier family 18 (vesicular monoamine transporter), member 1 (SLC18A1) and cytotoxic T-lymphocyte-associated protein 4 (CTLA4) have been previously implicated in SZ.

Vesicular monoamine transporters (VMATs) are involved in the packaging of dopamine, serotonin, adrenalin, and noradrenalin from the cytoplasm to their storage vesicles in presynaptic terminals. Dysregulation of monoamines has been long postulated to play an important role in the etiology of SZ and other neuropsychiatric disorders. SLC18A1 is one of the two isoforms of VMATs, playing a critical role in the maintenance of monoaminergic endocrine systems. SLC18A1 is mapped to chromosome 8p21.3, a locus with strong evidence for linkage with SZ. SLC18A1 has been reported to be associated with SZ[^24-27^](#_ENREF_24). In this study, we detected an increased level of expression of SLC18A1 in SZ patients, providing further support for its possible involvement into SZ.

CTLA4 is involved in establishing and maintaining peripheral T-cell tolerance, which controls T-cell activation and reactivity. The CTLA4 gene is located on chromosome 2q33, and has been suggested as a candidate gene for conferring susceptibility to autoimmune disease[^28^](#_ENREF_28). Numerous studies have demonstrated that SZ patients showed a T-cell-associated dysfunction[^29^](#_ENREF_29). Several studies have reported the association of CTLA4 variants with SZ[^30-33^](#_ENREF_30). Furthermore, significantly increased expression of CTLA4 was observed[^32^](#_ENREF_32), consistent with the results from our study.

DCEA identified two modules relevant to SZ. The green module is most striking, in that it has a high correlation with the disease, and involves most up-regulated genes (67.1%). This observation further suggested that most DE genes may be functionally related. The red module is an additional finding coming from the coexpression analysis. Although most genes within the red module are down-regulated, none of them reached the genome-wide significance level. These genes were missed by traditional differential expression analysis. However, these genes may also contribute to the development of the disease. The green and the red modules involve two distinctive clusters of genes with opposite expression trends and therefor association directions. Nonetheless, the intensive PPIs among the genes within two modules indicate that the two modules are interconnected and act jointly.

Coexpression modules highlight several hub genes, including CCDC134, UBD, C10orf67, TNIP3, and ZIC2, while PPI analysis indicates AKT1, CHEK1 and BRCA1 as hub genes. All of these hub genes are expressed in brain (Supplementary Figure S6). Since the majority of other genes are interacted with these hub genes, we postulate that most genes in the PPI network should be expressed in brain too. Among the hub genes, ZIC2, AKT1, CHEK1, and BRCA1 have been implicated in SZ, while CCDC134, UBD, C10orf67, and TNIP3 may be promising biomarkers warrant further investigation.

ZIC2 is a member of the ZIC family of zinc finger proteins, and functions as a transcriptional repressor with critical roles in neural development[^34^](#_ENREF_34). It has been suggested that the mice heterozygous for the hypomorphic mutation in Zic2 mimic the SZ-like phenotypes and ZIC2 mutations were associated with SZ. ZIC2 inhibits Wnt/β-catenin protein signaling pathway that is relevant to the pathogenesis of SZ. However, no significant difference between SZ cases and controls was observed in both our microarray dataset (FC = 0.43, P_Bonferroni_ = 1.00) and the RT-PCR analysis (FC = 0.13, FDR = 0.074).

A common function among CHEK1, BRCA1, and AKT1 is that they are relevant to cell cycle. CHEK1 and BRCA1 have been reported to be downregulated in SZ[^39^](#_ENREF_39). The impairment of AKT/GSK3 signaling pathway has been implicated in SZ and decreased AKT1 protein levels and phosphorylation have been observed in lymphocytes and brains of individuals with SZ. Several studies also detected the association of AKT1 genetic variants with SZ (reviewed in[^42^](#_ENREF_42)).

The expression levels of AKT1, BRCA1, CCDC134, UBD, and ZIC2 were validated in the RT-PCR analysis, with BRCA1 and AKT1 being upregulated in the patients; while after the 12-week treatment, the other three genes, CCDC134, UBD, and ZIC2 were slightly down-regulated. This result indicated that these genes may be trait-relevant rather than state-relevant.

Although there was a paucity of overlaps of the 84 DE genes with well-known candidate genes for SZ, several enriched pathways in our mRNA dataset have been implicated in SZ, including the p53 signaling pathway, the chemokine signaling pathway, and the MAPK signaling pathway[^45^](#_ENREF_45). Interestingly, a new pathway, the olfactory transduction pathway, was identified in the 446 module genes in our study. Impairments of olfactory system in schizophrenia patients have been well-documented. It has been suggested that dysfunction in the central and peripheral olfactory system in SZ is ubiquitous, which may be a useful marker of SZ[^47^](#_ENREF_47). Our study seemed to support the alteration of olfactory pathways in the blood of SZ patients.

Several limitations in this study need to be noted. Given the rarity of drug-naïve patients with secure diagnoses of EOS, the sample in the expression experiments was relatively small. A caveat in mind was that the mRNA results were derived from blood sample, thus, due care should be taken when extrapolating these results into the brain. It has been suggested that peripheral biomarkers may in part mirror similar pathological processes in the brain, in part represent a distinct molecular changes in blood that is highly specific to the primary pathophysiology, or reflect the molecular response of the peripheral cells secondary to the disease[^48^](#_ENREF_48). Thus, abnormalities in blood, whether or not they are mimicked in brain, are potential indicators of disease pathology.

Our study identified two clusters of SZ associated coexpressed modules, which may help unravel mechanisms underlying the disease. Several genes within the modules may be novel promising biomarkers for SZ.

**Methods**

**Subjects**

All participants were unrelated Han Chinese recruited from the north of China. Consensus diagnoses were made by at least two experienced psychiatrists independently according to the Diagnosis and Statistical Manual of Mental Disorders Fourth Edition (DSM-IV) criteria for SZ. Patients with unanimous diagnosis were enrolled into the study.

In the microarray analysis, samples included 18 first-onset SZ patients (8 males and 10 females, aged 14.78±1.70 years ranging from 10-18 years), and 12 healthy controls (6 males and 6 females, aged 14.75 ± 2.14 years ranging from 10-17 years). Healthy teenager controls were recruited from several middle schools with an interview conducted by psychiatrists; individuals with a history of mental health or neurological diseases were all excluded.

For the real-time quantitative PCR (RT-qPCR) analysis, we enrolled 38 SZ patients who were drug-free for at least one month before the enrollment; among them 30 patients (14 males and 16 females, aged 34.5±11.0 years) were successfully followed-up with a 12-week period of antipsychotic treatment. The clinical effects were assessed by trained and experienced psychiatrists with the Positive and Negative Syndrome Scale (PANSS) respectively before and after 12-week treatment. All patients got clinical improvement according to the PANSS reductive ratio more than 25%. All patients participating in the study were treated with one of the oral second generation or atypical antipsychotics (SGA) and tracked for 12-week continuous medication after baseline assessments. A total of 48 healthy controls (17 males and 31 females, aged 31.6±6.88 years) were recruited from local communities or were undergoing routine health check-ups. Subjects with relevant physical diseases or a history of major psychiatric disorders or suicidal behavior were excluded, and those who had a ﬁrst-degree relative with a history of severe mental disorder or suicidal behavior were also excluded.

There was no significant difference in gender or age between SZ cases and controls in the two cohorts of samples (Supplementary Table S8). Total RNA was isolated from peripheral blood mononuclear cells (PBMCs) using TRIzol (Invitrogen; USA) with on-column DNase I treatment as described by the manufacturer. Blood samples of each participant were collected on early morning before breakfast. Following the diagnosis, blood samples of the schizophrenia patients were collected next morning.

The study was approved by Medical Research Ethics Committee of Shanxi Medical University, and all experiments were performed in accordance with the approved guidelines and regulations. Informed consent was signed by both the teenage participants and their parents or care takers.

**mRNA profiling**

Total RNA was isolated from peripheral blood mononuclear cells (PBMCs) using TRIzol (Invitrogen; USA) with on-column DNase I treatment as described by the manufacturer. The concentration and purity of isolated RNA can be measured with the NanoDrop nd-1000 UV-Vis spectrophotometer. The concentration of isolated RNA was more than 1000ng/ul, the purity of RNA were both OD260/280 and OD260/230 very closed to 2.0. The intensity of these rRNA bands on denaturing agarose gels have been used to calculate a ratio that served as an indication of RNA integrity. A 28S/18S ratio of two indicates a good quality of RNA.

Agilent Array platform was employed to perform the microarray analysis. The sample preparation and microarray hybridization were performed based upon the manufacturer’s standard protocols with minor modifications. Briefly, mRNA was purified from total RNA after removal of rRNA (mRNA-ONLY™ Eukaryotic mRNA Isolation Kit, Epicentre). Then, each sample was amplified and transcribed into fluorescent cRNA along the entire length of the transcripts without 3’ bias utilizing a random priming method. The labeled cRNAs were hybridized onto the Human LncRNA Array v2.0 (8 x 60K, Arraystar). After having washed the slides, the arrays were scanned by the Agilent Scanner G2505B. Agilent Feature Extraction software (v10.7.3.1) was used to analyze acquired array images. Raw signal intensities were normalized in quantile method using GeneSpring GX v11.5.1 (Agilent Technologies), and low intensity mRNAs were filtered (mRNAs that at least 20 out of 30 samples have flags in Present or Marginal were chosen for further analysis). After normalization, the distributions of log2-ratios among all samples were nearly the same (Supplementary Figure S7). The raw microarray data is deposited at gene expression omnibus (GEO) and are accessible through GEO Series accession number GSE54913.

A total of 17219 valid probes were detected. Data on the probe level was collapsed to genes for separate datasets prior to analysis. When two or more probes were available (this is the case for most genes), expression levels of the probes were averaged to represent the expression level of the gene. This resulted in 8594 genes. Mis-mapped genes between gene symbols and Entrez IDs were filtered, resulting in 8518 genes.

**Analysis of gene expression by RT-qPCR**

cDNA was synthesized using High Capacity RNA-to-cDNA Kit (Invitrogen; USA) as described by the manufacturer. The primers were listed in Supplementary Table S9. PCR was performed using a ViiA 7 Real-time PCR System (Applied Biosystems) for 10 min at 95 °C, and then 40 cycles consisting of 10 s at 95 °C, 60 s at 60 °C, 15 s at 95 °C, followed by a subsequent standard dissociation protocol to ensure that each amplicon was a single product. All quantifications were normalized to GAPDH.

**Statistical analysis and in silico prediction**

R[^49^](#_ENREF_49) was used to perform the data processing and analyses of mRNA data. Student’s t-test was used to compare expression levels between the patient and control groups. Power of the gene expression data was estimated using R package ssize.fdr. Clustering heat maps were plotted with gplots[^52^](#_ENREF_52). Gene expression data were filtered using R package genefilter[^53^](#_ENREF_53). Gene ontology (GO) analysis was performed using R package piano [^54^](#_ENREF_54), and the minimum gene set size (number of member genes) was set to 5. KEGG pathway analysis was performed using R package GeneAnswers, and GO pie chart was plotted using R packages geneListPie[^57^](#_ENREF_57). Protein-protein interaction (PPI) network was retrieved via STRING v9.1[^58^](#_ENREF_58). The networks were plotted using Cytoscape[^59^](#_ENREF_59).

Coexpression analysis was conducted using R package WGCNA v1.43. A weighted adjacency matrix containing pair-wise connection strengths was constructed by using the soft-thresholding approach (β = 30, Supplementary Figure S8) on the matrix of pairwise correlation coefficients. A connectivity measure (k) per gene was calculated by summing the connection strengths with other genes. Modules are defined as clusters of densely interconnected genes; by default, they are indicated by branches of a hierarchical clustering tree using a dissimilarity measure. Then, each module is subsequently assigned a color. The gene expression profiles of each module were summarized by the module eigengene (defined as the first principal component of the module expression levels). A measure of gene significance (GS) is computed to evaluate a gene’s correlation with a phenotype. The intra-modular connectivity (k-within) was calculated for each gene by summing the connection strengths with other module genes and dividing this number by the maximum intra-modular connectivity. The adjacency threshold for including edges in the output was set at 0.2. Eigengene-based connectivity of a gene in a module is defined as the correlation of the gene with the corresponding module eigengene.

For RT-qPCR analysis, the comparative Ct (2^-ΔΔCT^) method was used for the quantification of transcripts. The differences of mRNA levels between cases and controls were analyzed using the Wilcoxon rank-sum test, and the differences of mRNA levels between schizophrenia cases before and after the treatment were analyzed using the paired Wilcoxon rank-sum test. The significance threshold was set at false discovery rate (FDR) < 0.05.

**Acknowledgements**

We sincerely thank all the subjects for their support and participation and all the medical staff involved in collecting blood samples. We thank Chunyu Liu and Simone de Jong for their help in the manuscript writing. This work was supported by the National Natural Science Foundation of China (81471364, 81271482, 81571319), Beijing Natural Science Foundation (7132182), Program for New Century Excellent Talents in University (NCET-12-1036). Shugart was supported by the Intramural Research Program of National Institute of Mental Health, National Institutes of Health (MH002929-05).

**Author contributions**

Y.X. and F.Z. designed the study; F.Z., W.Y. and Y.Y.S. did the statistical analysis and wrote the manuscript; G.W., K.Z., C.J., J.W., H.Y. and D.Z. contributed to administrative and material support.

**Additional information**

Supplementary information accompanies this paper at http://www.nature.com/scientificreports

Competing financial interests: The authors declare no competing financial interests.

**References**

Table 1 Association of module eigengene with disease

| Module | Genes |  | Coefficient | P |
| --- | --- | --- | --- | --- |
| green | 238 |  | 0.766 | 8.28E-7 |
| red | 208 |  | -0.579 | 8.01E-4 |
| turquoise | 578 |  | -0.345 | 0.062 |
| brown | 316 |  | -0.200 | 0.290 |
| blue | 372 |  | 0.062 | 0.747 |
| yellow | 259 |  | -0.037 | 0.845 |
| grey | 513 |  | 0.009 | 0.962 |

**Figure 1 | Volcano plot of mRNA expressions.** Plotted along the x-axis is the mean of log_2_ fold-change, along the y-axis the negative logarithm of the p-values. Red denotes the 82 up-regulated genes, blue the 2 down-regulated genes. The horizontal green line is the negative logarithm the Bonferroni-adjusted P-value threshold (5.82E-6).

**Figure 2** **| Pathway analysis of the genes.** Gene sets that are significant (p<0.001) in the non-directional class from the GO enrichment analysis. Only gene sets with 5 or more genes were retained. The network plot shows the relation between gene sets by connecting those that share member genes. The color of nodes indicates P values. The thickness of the edges corresponds to the number of shared genes. The size of the nodes corresponds to the size of the gene sets.

**Figure 3 | Hierarchical cluster tree of the 2484 genes.** The color bands provide a simple visual comparison of module assignments (branch cuttings) based on the dynamic hybrid branch cutting method. The first band shows the results from the automatic single block analysis and the second color band visualizes the gene significance measure: “red” indicates a negative correlation with disease, and “blue” indicates a positive correlation.

**Figure 4 | Intra-modular connectivity of the green and red modules.** Green nodes denote genes in the green module; red nodes denote red module genes. Triangle nodes with blue labels are transcriptional factors.

**Figure 5 | PPI and pathway analysis of the genes within the green and red modules.** (A) PPIs among the module genes (networks with nodes > 4 were included). Green nodes denote genes in the green module; red nodes denote red module genes. Triangle nodes with blue labels are transcriptional factors. (B) Enriched KEGG pathways of the module genes. Red nodes denote up-regulated genes, while blue nodes denote down-regulated genes.

**Figure 6 | Expression levlels (log2 transformed) of several key genes via RT-qPCR analysis.** HC: healthy control; SZ: untreated schizophrenia patients; SZ_12w: schizophrenia patients after 12-week treatment. Ventical bars denote 95% confidence interval of expressin levels for each gene.
